# Structural Analysis and Aggregation Propensity of Pyroglutamate Aβ(3-40) in Aqueous Trifluoroethanol

**DOI:** 10.1371/journal.pone.0143647

**Published:** 2015-11-23

**Authors:** Christina Dammers, Lothar Gremer, Kerstin Reiß, Antonia N. Klein, Philipp Neudecker, Rudolf Hartmann, Na Sun, Hans-Ulrich Demuth, Melanie Schwarten, Dieter Willbold

**Affiliations:** 1 Institute of Complex Systems (ICS-6) Structural Biochemistry, Forschungszentrum Jülich, 52425, Jülich, Germany; 2 Institut für Physikalische Biologie, Heinrich-Heine-Universität Düsseldorf, 40225, Düsseldorf, Germany; 3 Fraunhofer Institute for Cell Therapy and Immunology, Department of Drug Design and Target Validation, 06120, Halle (Saale), Germany; Nanyang Technological University, SINGAPORE

## Abstract

A hallmark of Alzheimer’s disease (AD) is the accumulation of extracellular amyloid-β (Aβ) plaques in the brains of patients. N-terminally truncated pyroglutamate-modified Aβ (pEAβ) has been described as a major compound of Aβ species in senile plaques. pEAβ is more resistant to degradation, shows higher toxicity and has increased aggregation propensity and β-sheet stabilization compared to non-modified Aβ. Here we characterized recombinant pEAβ(3–40) in aqueous trifluoroethanol (TFE) solution regarding its aggregation propensity and structural changes in comparison to its non-pyroglutamate-modified variant Aβ(1–40). Secondary structure analysis by circular dichroism spectroscopy suggests that pEAβ(3–40) shows an increased tendency to form β-sheet-rich structures in 20% TFE containing solutions where Aβ(1–40) forms α-helices. Aggregation kinetics of pEAβ(3–40) in the presence of 20% TFE monitored by thioflavin-T (ThT) assay showed a typical sigmoidal aggregation in contrast to Aβ(1–40), which lacks ThT positive structures under the same conditions. Transmission electron microscopy confirms that pEAβ(3–40) aggregated to large fibrils and high molecular weight aggregates in spite of the presence of the helix stabilizing co-solvent TFE. High resolution NMR spectroscopy of recombinantly produced and uniformly isotope labeled [*U*-^15^N]-pEAβ(3–40) in TFE containing solutions indicates that the pyroglutamate formation affects significantly the N-terminal region, which in turn leads to decreased monomer stability and increased aggregation propensity.

## Introduction

The pathology of Alzheimer’s disease (AD) is characterized by the presence of intracellular neurofibrillary tangles consisting of accumulated hyperphosphorylated tau protein and extracellular plaques containing amyloid-β (Aβ) as major component [1, 2]. Aβ is derived by processing of the amyloid precursor protein by β- and γ-secretases [3, 4]. Multiple possible cleavage positions of γ-secretase combined with various posttranslational modifications of the cleaved products result in different Aβ isoforms with heterogeneity in length as well as N- and C-terminal sequences [5, 6]. Pyroglutamate-modified Aβ (pEAβ) peptides have been reported to be the dominant component of all N-terminal truncated Aβ variants in AD plaques as up to 20% of the total Aβ are pEAβ variants [7–9]. pEAβ deposits in the brains of presymptomatic AD patients [10] and was shown to play a dominant role in plaque formation and to provoke neurodegeneration in mouse models [11, 12]. Formation of pEAβ is based on N-terminal truncation leading to N-terminal E3 or E11 and subsequent cyclization of the accessible E amino group with the side chain carboxy group leading to pyroglutamate. *In vivo* it was suggested the cyclization is catalyzed by the enzyme glutaminyl cyclase [13, 14]. pEAβ starting at position 3 (pEAβ(3-x)) represents the major fraction of N-pyroglutamate modified Aβ species in intracellular, extracellular and vascular accumulations in AD brains [15–18]. The cumulative deposition of pEAβ in AD brains coincides with increased protease-stability since the pyroglutamate modified N-terminus is inaccessible to aminopeptidases [19, 20]. The loss of two negative charges (side chain carboxyl groups of D1 and E3) and one positive charge (E3 amino group) at physiological pH result in higher hydrophobicity of pEAβ(3-x) [19, 21]. Aggregation of pEAβ(3-x) is up to 250-fold accelerated [22] and its neurotoxicity is enhanced [23, 24] when compared with the corresponding wild type Aβ species independent of the C-terminal length.

Structural analysis of pEAβ monomers is essential to uncover its aggregation mechanism and to reveal, why it is more prone to self-assembly than Aβ wild type. In a previous NMR study, we analyzed pEAβ(3–40) in aqueous buffer conditions at neutral pH and compared it with given Aβ(1–40) data [25]. Most chemical shifts were nearly identical except of the very N-terminal region and both peptides were shown to be unstructured.

Since structural studies of Aβ peptides are often limited due to their high aggregation tendency, solvent conditions need to be carefully chosen. α-helical Aβ monomers are predominantly formed in micelles [26], hexafluoroisopropanol (HFIP)/water [27] and 2,2,2-trifluoroethanol (TFE)/water [28] mixtures. In a previous NMR study we compared pEAβ(3–40) with Aβ(1–40), both chemically synthesized with natural carbon and nitrogen isotope abundance, in aqueous solution containing 40% TFE [29]. The results indicated that both Aβ isoforms formed α-helical structures in two regions (aa 14–22 and aa 30–36) connected by a flexible and disordered linker. However, pEAβ(3–40) exhibited a decreased helix propensity in agreement with its higher hydrophobicity and faster aggregation kinetics.

Here, we analyzed the solvent conditions for the transition of pEAβ(3–40) from TFE-induced α-helices to β-sheets by gradually lowering the TFE concentrations. We expand the data by investigations of recombinantly produced [*U*-^15^N]-pEAβ(3–40) regarding structure and aggregation in 20% TFE, where Aβ(1–40) is still in a predominantly α-helical monomeric state, while pEAβ(3–40) forms a β-sheet dominated secondary structure, starts to aggregate and forms large fibrils.

## Materials and Methods

### Peptides and sample preparation

Expression and purification of uniformly isotope labeled [*U*-^15^N]-pEAβ(3–40) and [*U*-^15^N]-Aβ(1–40) as well as non-labeled pEAβ(3–40) were performed as described recently [25, 30]. Synthetic non-labeled Aβ(1–40) was purchased from Bachem (Heidelberg, Germany). Samples were prepared in Protein LowBinding tubes (Eppendorf AG, 230 Hamburg, Germany). The Aβ peptides were dissolved in HFIP (1,1,1,3,3,3,-hexafluoro-2-propanol, Sigma-Aldrich, Hannover, Germany) and monomerized for 3 days at room temperature. Samples were lyophilized and stored at -20°C.

### CD spectroscopy

Lyophilized peptides were dissolved in aqueous buffer containing different TFE concentrations (20–25% TFE in 50 mM potassium phosphate, pH 2.8) to a final concentration of 25 μM. UV CD measurements were performed in 1 mm path-length cuvettes at 20°C. CD spectra were recorded on a Jasco J-1100 spectropolarimeter from 260 to 187 nm with 0.5 nm step size, 50 nm/min scan speed, 1 nm bandwidth and 10 scans per sample. Background correction was performed by subtraction of corresponding buffer spectra.

### ThT assay

Lyophilized peptides were dissolved to a final concentration of 25 μM in buffer (20–25% TFE in 50 mM potassium phosphate, pH 2.8) containing 10 μM ThT. Aggregation assays were performed in black non-binding 96-well plates (Sigma-Aldrich, Germany) at 20°C with a total volume of 100 μl per well. Each reaction was performed fivefold and background corrected by subtraction of the buffer control. Fluorescence was monitored in 10 min steps using a microplate reader (PolarStar Optima, BMG, Offenburg, Germany) with 440 excitation and 492 nm emission filters, respectively, in bottom-read mode. Wells were shaken 30°s prior to measurement.

### TEM microscopy

Lyophilized pEAβ(3–40) was dissolved in aqueous buffer (20% TFE in 50 mM potassium phosphate, pH 2.8) and incubated for five days at 20°C. Fibrils were absorbed on formval/carbon coated copper grids (S162, Plano, Wetzlar, Germany) for 5 min and washed with water. Negative staining was performed by incubation with 1% (w/v) uranylacetate for 1 min. Images were taken with a Libra 120 transmission electron microscope (Zeiss, Oberkochen, Germany) at 120 kV.

### NMR spectroscopy

Lyophilized [*U*-^15^N]-pEAβ(3–40) and [*U*-^15^N]-Aβ(1–40) were directly dissolved in 100% TFE-d2-OH and diluted with 50 mM potassium phosphate, pH 2.8 to a final concentration of 25 μM peptide in 20% TFE. NMR spectra were acquired using a TXI- or QCI-cryoprobe equipped Bruker Avance III HD 600 MHz spectrometer. ^1^H,^15^N-HSQC correlation spectra were recorded according to standard Bruker pulse-sequences [31–33]. ^1^H_N_ and ^15^N Backbone resonance assignments in 30% TFE were obtained by using BEST-TROSY (BT) HNCA+ optimized pulse sequences [34, 35]. Resonance assignments of spectra recorded at 30% TFE concentration were transferred to the spectrum recorded at 20% TFE through recordings at TFE concentrations of 27% and 23%, respectively. Spectra were processed with NMRPipe [36] and evaluated with CCPNmr Analysis [37]. Analysis of Cα secondary chemical shifts is based on the publication by Zhang *et al*. [38] and sequence corrected according to Schwarzinger [39].

## Results

### Secondary structure analysis by CD spectroscopy

**Fig 1:**
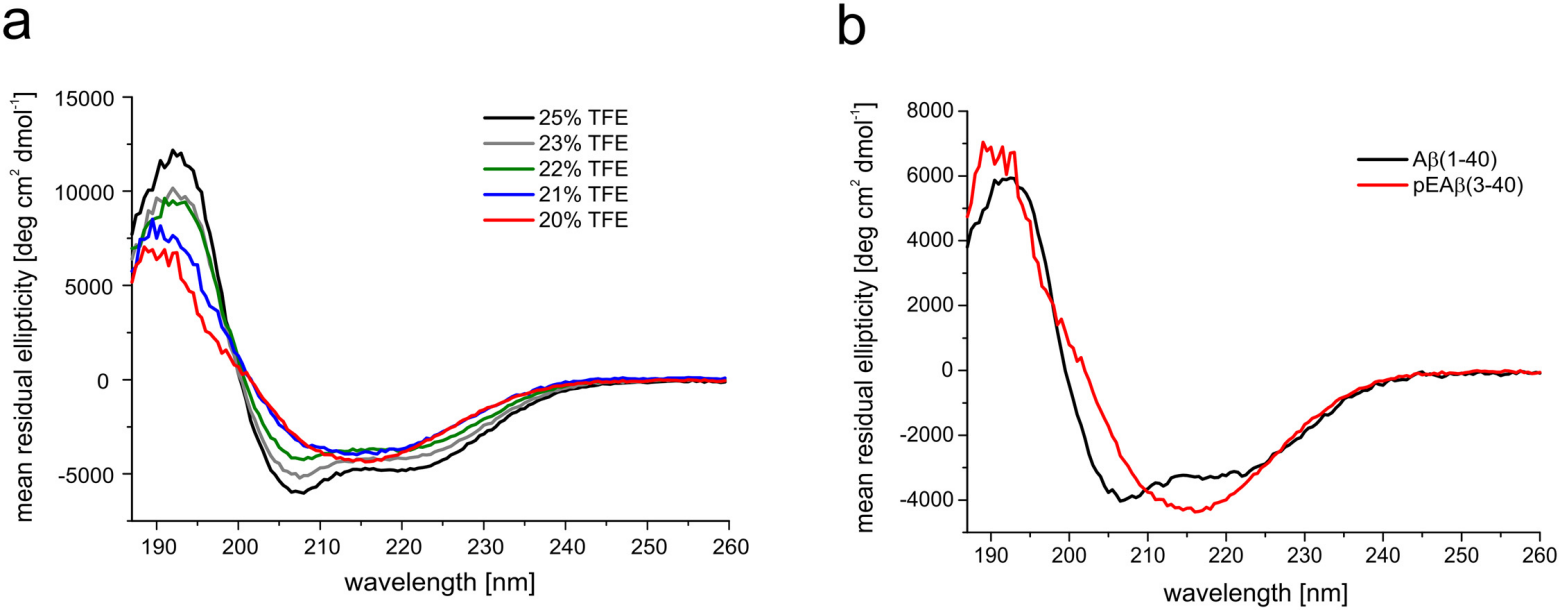
Far-UV CD spectra of pEAβ(3–40) and Aβ(1–40). Peptides were dissolved in buffer (20–25% TFE in 50 mM potassium phosphate, pH 2.8). CD spectra were recorded at 20°C from 260 to 187 nm, accumulated 10 times and background corrected. (a) CD spectra of 25 μM pEAβ(3–40) in 25%, 23%, 22%, 21% and 20% TFE showed a shift from α-helical structure towards β-sheets with decreasing TFE concentrations. (b) The CD spectrum of 25 μM Aβ(1–40) indicated α-helices in 20% TFE while the spectrum of 25 μM pEAβ(3–40) in 20% TFE showed mainly β-sheet rich structures.

TFE is known to induce α-helical structures by lowering the solvent polarity and promoting intra-molecular hydrogen bonding [40]. Reducing the concentration of this co-solvent prevents this effect e.g. as shown for Aβ(1–40) [41], human carbonic anhydrase II [42], FF domain of URN1 splicing factor [43], transferrin [44], α-synuclein [45] and conalbumin [46]. The α-helices inducing effect of TFE on pEAβ(3–40) and the shift towards β-sheets by lowering the TFE contents was analyzed by recording CD spectra in aqueous buffer in different TFE concentrations. CD spectra in solutions containing varying concentrations of TFE ranging from 25% to 22% at pH 2.8 indicated a mainly α-helical conformation based on minima at 208 nm and 222 nm, a maximum at 193 nm and an x-axis intercept at 200 nm. Marginally loweringthe TFE concentration from 22% to 20% or 21% was sufficient to alter the α-helical protein conformation of pEAβ(3–40) to a β-sheet dominated secondary structure (Fig 1a) as indicated by the evolvement of characteristic β-sheet features in the corresponding CD spectra with a single minimum at 216 nm, a x-axis intercept at 202 nm and a shift of the maximum to a lower wavelength, i.e. 190 nm. CD spectra of Aβ(1–40) recorded under the same condition (20% TFE) differed compared to the spectra of pEAβ(3–40) by indicating α-helices (Fig 1b).

### Aggregation kinetics and fibrillation

**Fig 2:**
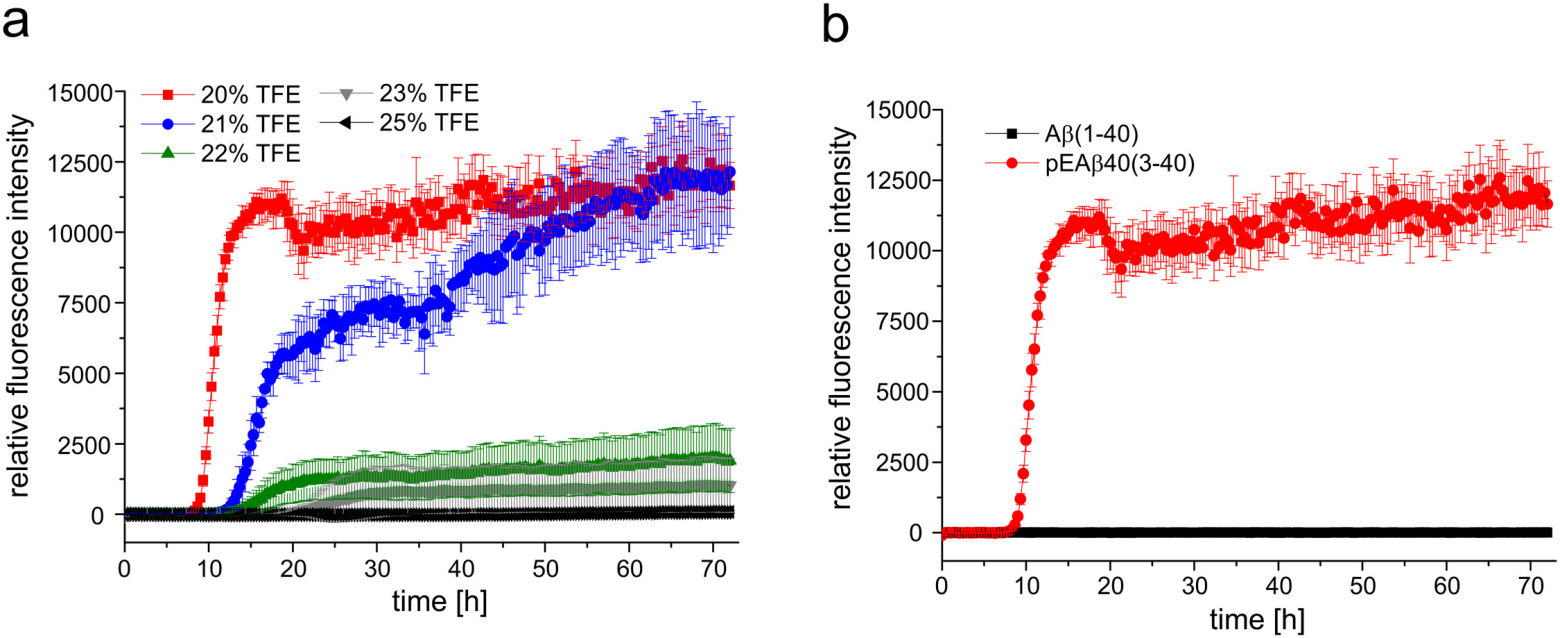
Aggregation kinetics of pEAβ(3–40) and Aβ(1–40) in TFE. (a) 25 μM of monomerized pEAβ(3–40) were dissolved in buffer with various TFE contents (25%, 23%, 22%, 21% and 20% TFE in 50 mM potassium phosphate, pH 2.8) including 10 μM ThT. pEAβ(3–40) aggregated in 20% and 21% TFE but was significantly decreased in aqueous solution with higher TFE concentration. (b) 25 μM of monomerized pEAβ(3–40) and Aβ(1–40) were dissolved in buffer (20% TFE in 50 mM potassium phosphate, pH 2.8) including 10 μM ThT. An increase in ThT fluorescence was observed for pEAβ(3–40) but not for Aβ(1–40) within 72 h.

Aggregation analysis supported the CD data of pEAβ(3–40) and Aβ(1–40) indicating different secondary structural elements in aqueous solution containing 20% TFE. Fig 2a displays the aggregation kinetics in TFE containing solutions ranging from 20% to 25% TFE at 20°C for pEAβ(3–40) as monitored by ThT assay. pEAβ(3–40) aggregation reaches its maximum at TFE concentrations of 20% and 21%. A distinct lag phase of 9h (20% TFE) or 14 h (21% TFE) is observable following a clear growth phase where aggregation increased indicating that β-sheet formation is not hindered by the presence of TFE. In the presence of 25% TFE no THT fluorescence increase was observed during 72 h, which indicate that pEAβ(3–40) stays monomeric at that condition. However, when the TFE concentration was lowered to 23% or 22% TFE a significant delay as well as decreased maximum was detected within 72 h exploration time. Aggregation of Aβ(1–40) could not be observed at all under the same conditions as no increase in fluorescence intensity was detected within 72 h (Fig 2b). Thus, fibrillation and/or ThT positive β-sheet formation of Aβ(1–40) seemed to be inhibited or at least significantly delayed in the presence of the helix-stabilizing fluoroalcohol TFE.

**Fig 3:**
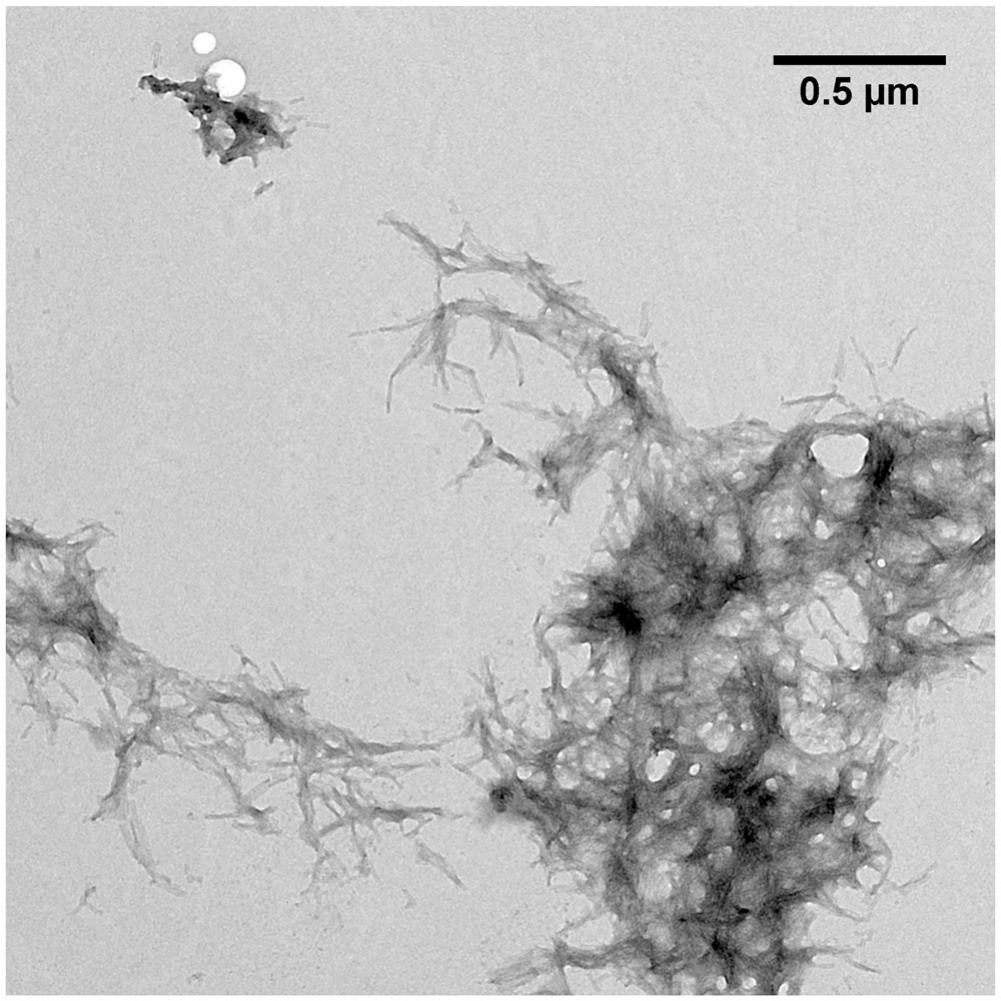
TEM image of pEAβ(3–40) in 50 mM potassium phosphate pH 2.8 containing 20% TFE. Monomerized pEAβ(3–40) (25 μM) was incubated for fibrillation at 20°C for five days and grids were prepared by negative staining. pEAβ(3–40) incubated in aqueous TFE solution formed large twisted fibrils up to several hundred nm in size which accumulate into large aggregates ranging from 1–5 μm in diameter.

TEM microscopy was performed to analyze the structure of pEAβ(3–40) aggregation products formed after incubation of the monomerized pepide in 20% TFE (Fig 3). pEAβ(3–40) built large fibrils accumulating into aggregates with several μm in diameter. The fibrils were twisted, exhibit a rather homogeneous morphology and accumulate into plaques comparable with fibrils matured in aqueous solution [47].

### NMR spectroscopy

Since pEAβ(3–40) started to aggregate at room temperature and built large fibrils in 20% TFE (pH 2.8), 2D NMR spectroscopy was performed with only 25 μM recombinantly produced [*U*-^15^N]-pEAβ(3–40). Nonetheless, considerably protein aggregation within 2 days even at the low concentration applied impeded the recording of 3D experiments. To overcome this problem, backbone ^1^H_N_ and ^15^N resonance assignments of pEAβ(3–40) in 20% TFE were obtained by a time optimized BT-HNCA+ pulse sequence performed with pEAβ(3–40) in 30% TFE (S1 Fig). Transfer of the resonance assignments to the ^1^H,^15^N-HSQC spectrum in 20% TFE was performed gradually from 30% through 27% and 23% TFE (S2 Fig). The Cα secondary chemical shifts of pEAβ(3–40) in 30% TFE were neighbor-corrected and plotted as a function of the aa sequence (S3 Fig). There is evidence that aa Y10-A21 and K28-V36 are involved in the α-helix formation as indicated by positive secondary chemical shifts. This is in accordance with the secondary structure of pEAβ(3–40) in 40%TFE obtained from proton chemical shift data [29].

**Fig 4:**
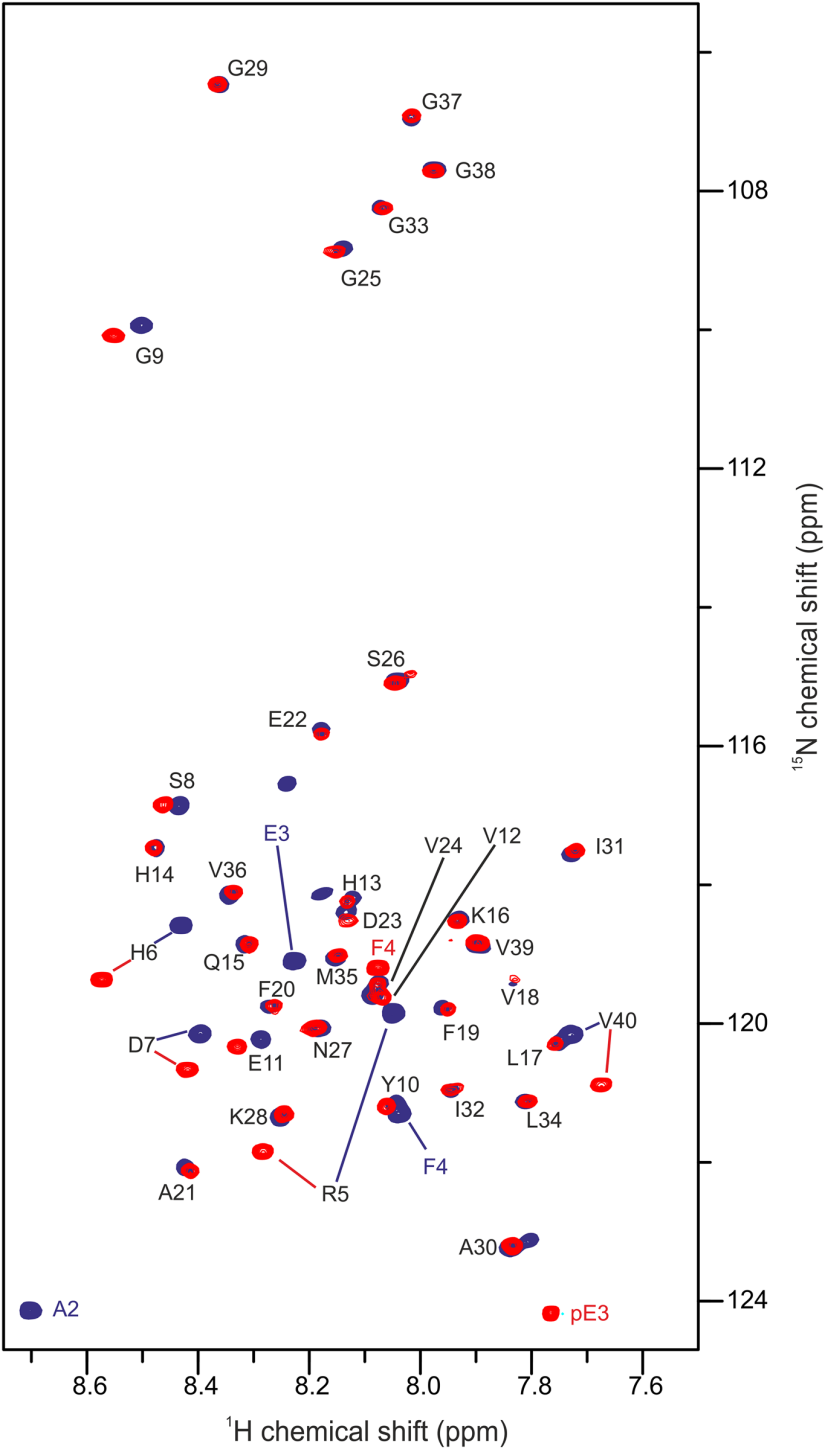
^1^H,^15^N-HSQC of pEAβ(3–40) and Aβ(1–40). 25 μM of the monomerized peptides were dissolved in aqueous solution (50 mM potassium phosphate, pH 2.8) containing 20% TFE. Spectra were recorded on a 600 MHz Bruker spectrometer at 5°C. Overlay of the spectrum of pEAβ(3–40) (red) and Aβ(1–40) (blue) indicate that the pyroglutamate modification affects the N-terminal signals significantly towards E11 as well as V40.

**Fig 5:**
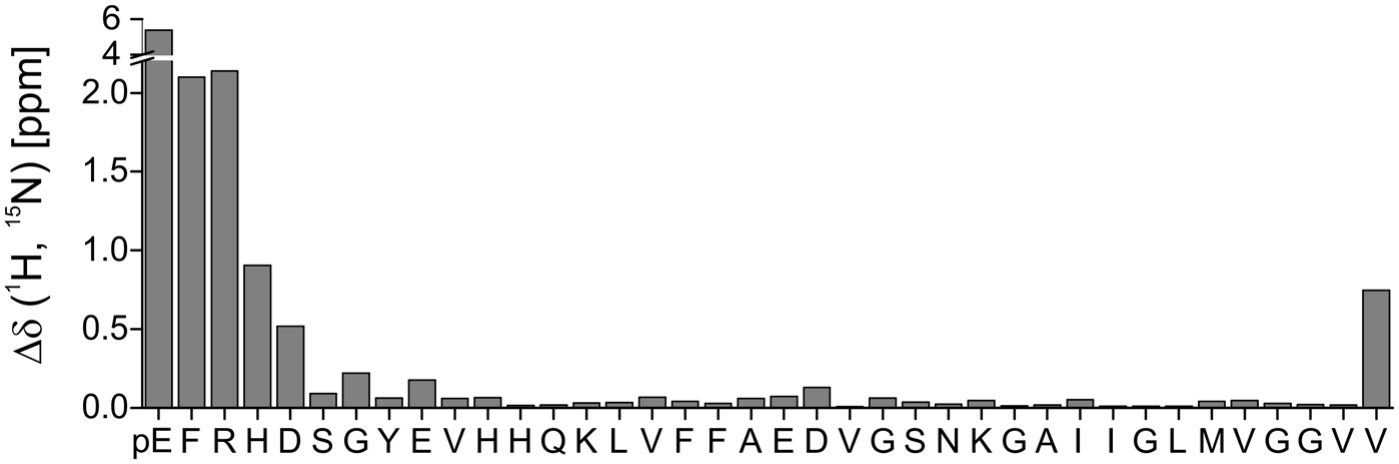
Chemical shift changes Δδ(^1^H,^15^N) = ((10*Δδ1H^N^)^2^ + (Δδ^15^N)^2^)^1/2^ between pEAβ(3–40) and Aβ(1–40) plotted as a function of the pEAβ(3–40) sequence. Pyroglutamate formation affects the N-terminal amino acids with decreasing effect towards the C-terminus and, interestingly, also the C-terminal V40.

For reference, the spectrum of recombinant [*U*-^15^N]-Aβ(1–40) was acquired under identical conditions and both ^1^H,^15^N-HSQC spectra were overlaid for comparison (Fig 4). The absence of the first two amino acid residues D1 and A2 and the substitution of E3 to pE3 is the only difference between pEAβ(3–40) and Aβ(1–40). The main changes in chemical shifts are therefore expected to be primarily observed for the very N-terminal amino acid residues. Interestingly, signals of the N-terminal region up to E11 differed significantly (Fig 5). Cross-peaks further downstream towards the C-terminus, except of the C-terminal amino acid V40, were not or only marginally changed.

## Discussion

Previous data indicated that pyroglutamate-modified Aβ shows increased tendency to form β-sheets compared to the unstructured Aβ in aqueous buffer at neutral pH [22, 48, 49]. Here, we expand structural knowledge on pEAβ(3–40) by showing that it forms β-sheets even in the presence of considerably amounts of the co-solvent TFE. This is consistent with data indicating that the tendency to build α-helical structures is decreased for pEAβ(3–40) as compared to unstructured Aβ(1–40) as analyzed by proton NMR spectroscopy at higher TFE concentrations, where both peptides stay in monomeric conformation [29]. Our data indicate a change in conformation of pEAβ(3–40) as a result of lowering the TFE concentration. We identified the exact TFE concentration (20% at pH 2.8) where pEAβ(3–40) already is in a β-sheet conformation, whereas Aβ(1–40) exhibits still an overall α-helical conformation. This confirms, that TFE is not simply favoring α-helical secondary structures, but secondary structures in general [50].

The differences in the secondary structure content observed by CD spectroscopy seem to be a result of the modification from Aβ to pEAβ, which modifies the N-terminal region significantly. ThT and TEM data support this evidence by yielding characteristic aggregation kinetics and large matured fibrils for pEAβ(3–40) in 20% TFE but not for Aβ(1–40) under same conditions.

Using NMR spectroscopy, we analyzed pEAβ(3–40) monomers in 20% TFE (pH 2.8). Under these conditions, pEAβ(3–40) slowly forms β-sheet containing aggregates that could not be observed by solution NMR. Although it was shown in a model peptide starting with a N-terminal E1 that conversion to pE1 results in altered chemical shifts only of the following two amino acids [51], pE formation of Aβ peptides obviously affects not only the immediately adjacent N-terminus. It was previously shown by H_N_ and H_α_ proton chemical shift differences between pEAβ(3–40) and Aβ(1–40) in higher TFE concentrations (40%), that mostly protons of the six following amino acids from pE3 to S8 with decreasing influence towards the C-terminus were affected [29]. In the presence of only 20% TFE, we observed many resonance differences of pEAβ(3–40) compared to Aβ(1–40). Chemical shift differences of the N-terminal amino acids towards E11 as well as the C-terminal V40 were mostly affected as shown in Fig 5. Thus, it seems that the modification of Aβ(1–40) to pEAβ(3–40) not only influence the neighboring amino acids, but also change significantly the conformational state of at least 25% of all amino acids. This result suggests that the N-terminal modification to pEAβ has a significant effect on secondary structure elements and thus is the driving force for pEAβ(3–40) to be more likely to build β-sheet structures under exactly the same conditions when compared to Aβ(1–40). The propensity of pEAβ(3–40) to aggregate in aqueous TFE solution seems to be propagated by the modified N-terminus, but V40 obviously play also a central role, maybe due to interactions of both termini. Since C-terminally truncated Aβ peptides are less prone to aggregation, e.g. Aβ(1–38) < Aβ(1–40) < Aβ(1–42) [52, 53], it is likely that their pE modified isoforms would show an increased aggregation propensity as compared to the non-pE-modified isoforms, analogous to the results obtained within this study.

## Supporting Information

S1 Fig ^15^N-HSQC of pEAβ(3–40) in 30% TFE. (TIF)

S2 Fig ^15^N-HSQC of pEAβ(3–40) in different TFE concentrations. (TIF)

S3 Fig Cα secondary chemical shifts of pEAβ(3–40) in 30% TFE. (TIF)
