# Supplementary figures and images for: Structural Analysis and Aggregation Propensity of Pyroglutamate Aβ(3-40) in Aqueous Trifluoroethanol
Source: PLoS One. 2015 Nov 23;10(11):e0143647. doi: 10.1371/journal.pone.0143647 (PMC4658145; doi:10.1371/journal.pone.0143647)

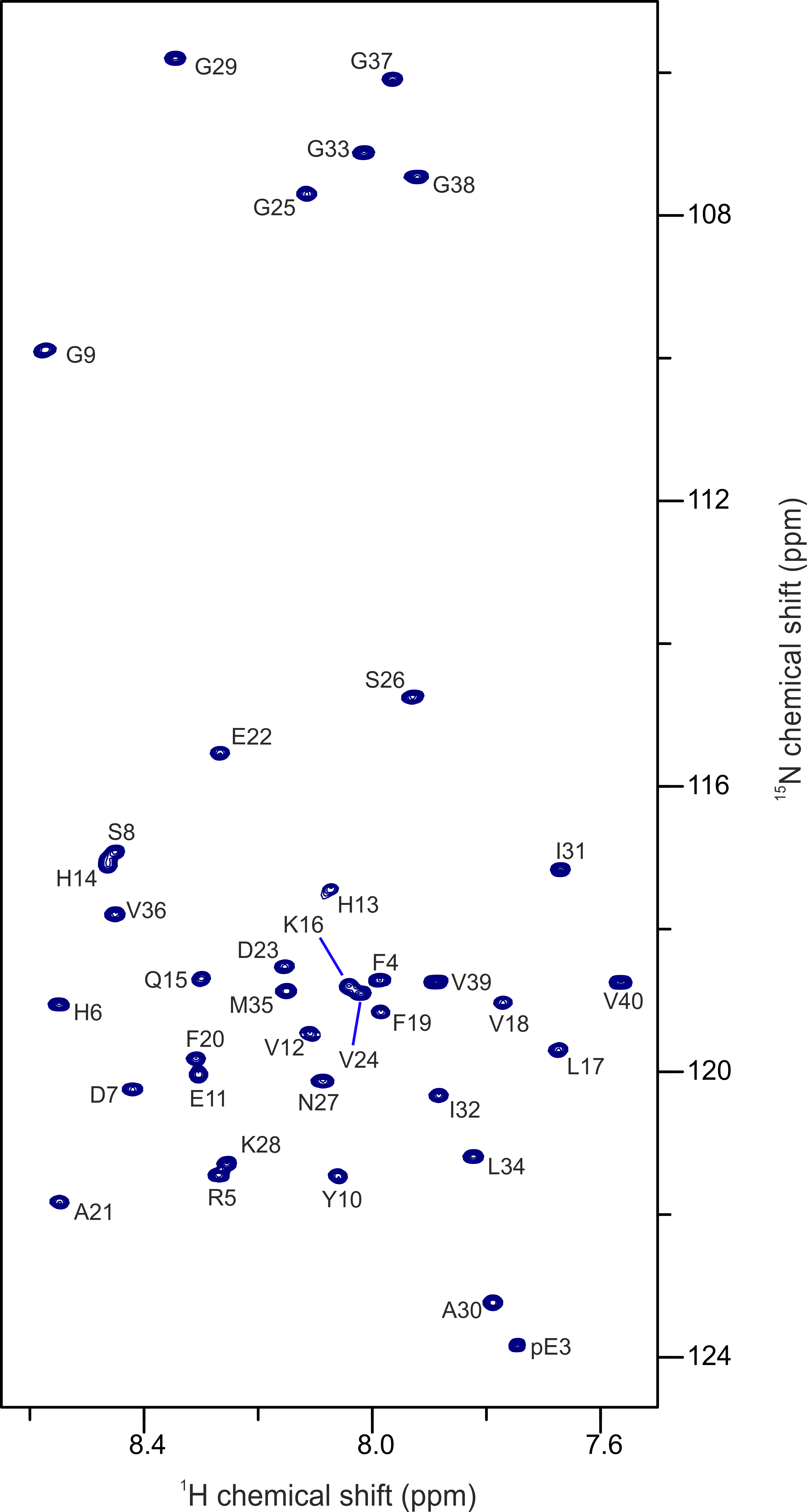

Supplement: S1 Fig — (TIF) [file pone.0143647.s001.tif]

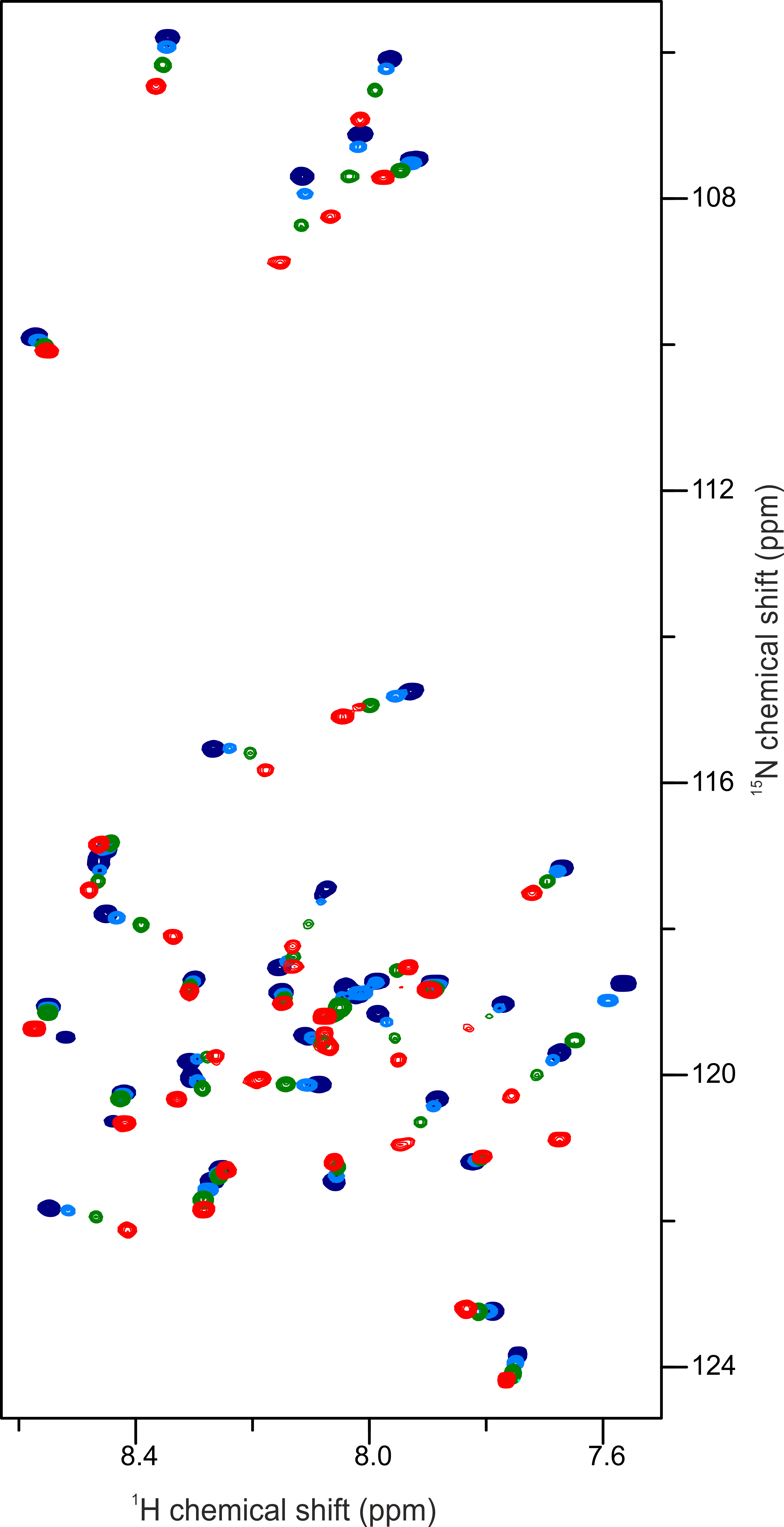

Supplement: S2 Fig — (TIF) [file pone.0143647.s002.tif]

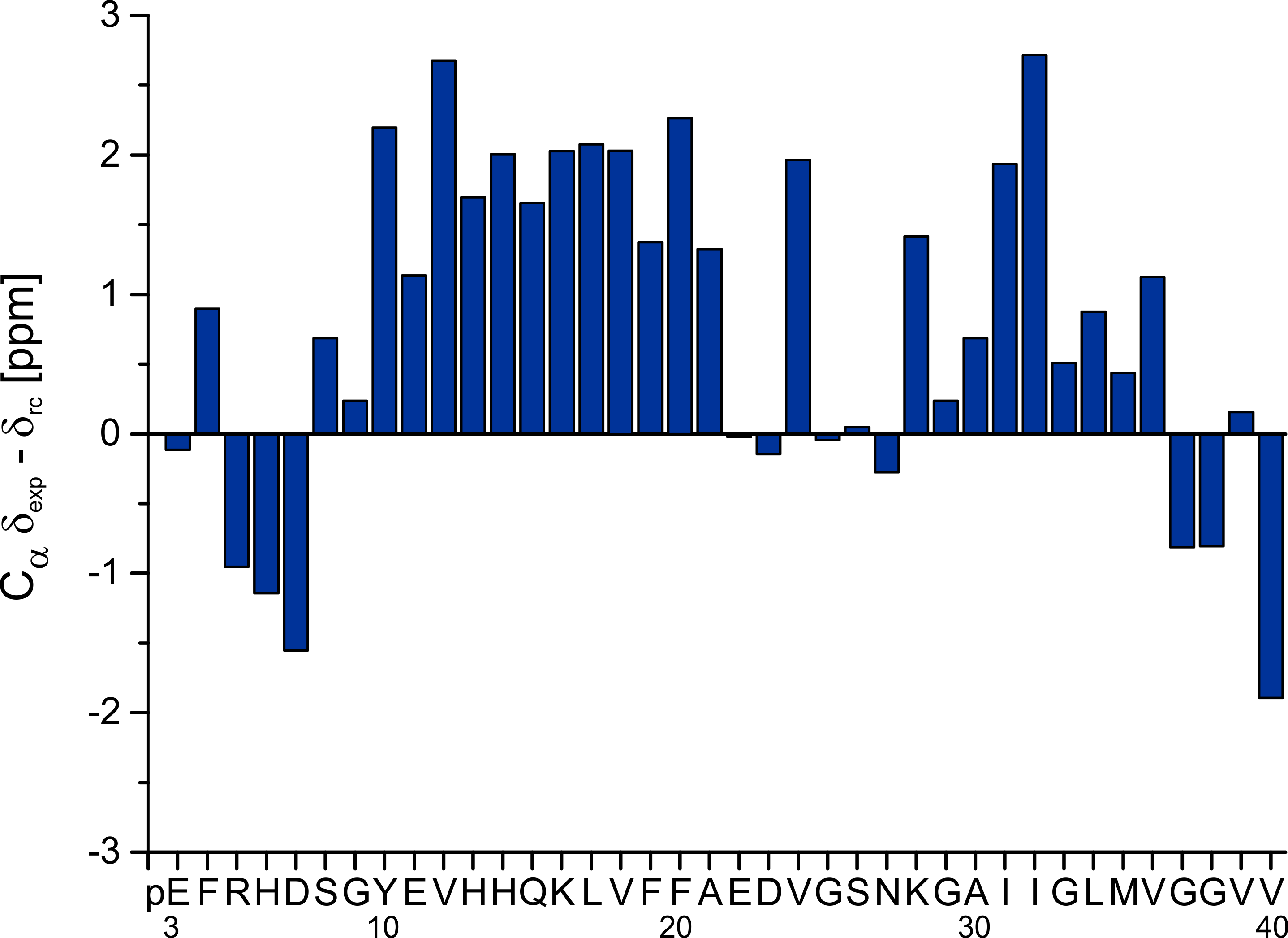

Supplement: S3 Fig — (TIF) [file pone.0143647.s003.tif]
